# Medical Statistics

**Published:** 1839

**Authors:** 


### MEDICAL STATISTICS.

I. Statistical Reports of the Sickness, Mortality, and Invaliding among the Troops in the United Kingdom, the Mediterranean, and British America; Prepared from the Records of the Army Medical Department and War-Office Returns.

II. First Annual Report of the Registrar-General of Births, Deaths, and Marriages in England. Octavo, pp. 168. London, 1839.

We are only beginning to apply the numerical method to medicine, and we can scarcely be said to have begun to derive any results from it. Yet valuable results will be obtained, perhaps very unexpected ones. It would be presumptuous to limit the use or the sphere of such an engine of exactness, because it is impossible to foresee all the improvements of which our science is susceptible. But liberal as we may be, and cautious as we must be, we cannot but deprecate the absurd lengths to which some enthusiasts have carried their admiration of medical statistics, and to which they have stretched its employment. Minds of this lob-sided construction there will always be; the mass of thinking men will take care not to lurch to leeward with them.

The object, at present, is to amass facts; the inferences will follow after. Each surgeon, however limited his sphere of practice, can help in this important work, and increase his own store of knowledge at the same time. And not a surgeon in England but should do so. Let him keep accurate memoranda of his cases—let him observe carefully, report correctly, and conclude without prejudice and without fear. If every medical man *would* do this, how changed would the face of medicine speedily be.

We turn to the works before us, and first to the Report of the Registrar-General.

*Success of the new System of Registration.*

It would appear that the registration is already answering. In spite of the opposition extended to it, in spite of the clamour raised and the difficulties thrown in its way, and in spite of the clumsiness with which new machinery must necessarily work at first, the system has, even in the first year, been eminently successful. To us, as medical men, and for the sake of medical science, this intelligence is cheering.

The Registrar informs us, *that*, It appears from the comparative account of the population of Great Britain in the years 1801, 1811, 1821, 1831, laid before Parliament in 1831, pursuant to the Act of 2 Geo. IV. c. 30, that the population of England and Wales had increased from 8,872,980 in 1801, to 10,163,676 in 1811, to 11,978,875 in 1821, and to 13,894,574 in 1831, the rate of increase, from 1801 to 1831, being 16·13 decennially; and from 1821 to 1831, being 16·01. Assuming that the latter rate of increase, which differs so slightly from that of the whole 30 years, has continued from 1831 to 1838, it will result from calculation that the population of England and Wales on January 1, 1838 (the middle of the first year of registration ending June 30, 1838,) was about 15,324,720.

It is assumed in the Preface to the Population Abstract for 1831, that the medium population for the years 1821 to 1830 was 12,938,030; and it appears from the same authority, and from the Abstracts of Answers and Returns under the Population Act 11 Geo. IV. c. 30, that the mean annual number of registered Burials during that period was 246,290. If the proportion which the registered Burials bore to the population in the years 1821 to 1830, is applied to the population in 1838, estimated to be about 15,324,720, it will appear that the probable number registered in the Parochial Registers for the year ending June 30, 1838, will have been about 291,715. But the number of Deaths registered under the new system, in the year ending June 30, 1838, has been 335,956, thus exceeding the estimated number in the Parochial Registers of Burials by 44,241.

"The greater efficacy," the Registrar goes on to observe, "of the new system in approximating to a complete Registration of Deaths, is sufficiently apparent from the foregoing statement; but it remains to be shown to what extent it is successful, and what is the whole number of Deaths which could have been registered if there had been absolutely no omissions. Here no proof can be afforded, and recourse must be had to calculation for an approximation to the probable truth. Reverting to the Preface of the Population Abstract for 1831, I find it stated that, upon the supposition that 'one-sixth may be justly added to the average of the registered Burials, (1820—1830,) and assuming the medium population at 12,938,030, the proportion of Deaths has been *one* in 45 of the population.' Apply this latter number as a divisor to the estimated population in the year ending June 1838, and the quotient, showing the probable number of Deaths in that year, will be 340,549. Mr. Finlaison, in his letter subjoined in the Appendix, has estimated the number of Deaths at 335,968.

Now the number actually registered has been 335,956, a number approximating very remarkably to that of Mr. Finlaison's estimate. It is evident, however, that this latter estimate is a little too low, for it must be remembered that the *first* year's Registration cannot comprise the deaths of the *whole* year; and that, while no deaths which occurred before July 1, 1837, can be included in the first year's returns, there will be many which occurred near the close of the fourth quarter ending June 30, 1838, which will not have been registered in that quarter, and can be included only in the Abstract of the year ending June 30, 1839. I have ascertained that 2,704 Deaths, which occurred in the first year of Registration, have been thus registered in the first quarter of the second year; therefore, the whole number of Deaths occurring in the first year of Registration, which have been registered then and afterwards, is at least 338,660, a number less than the highest of the two estimates by only 1,889, and affording strong presumptive evidence of a success in the practical operation of this part of the new system, which must probably have exceeded the most sanguine expectations that could have been entertained." 21.

*Registration of Births.*—This has not been so successful as the Registration of Deaths. Yet it has beat the old registry of Baptisms already, and will probably be more and more successful as prejudices wear away, opposition is tired out, and knowledge of the advantages of the measure makes progress.

### *Expectation of Life at various Ages.*

"I am aware that with a progressive population, and especially with a population of which the ratio of increase appears to have varied so considerably in different parts of the kingdom, (varying, between the years 1821 and 1831, from 2 per cent. in the North Riding of Yorkshire to 36 per cent. in Monmouthshire,) an attempt to form a Table of Mortality, showing the expectation of life at successive ages, deduced alone from the Abstracts of Deaths for a single year, must be attended with great risk of error. I shall, therefore not attempt to form any such table from such materials, but shall exhibit only recorded facts, accompanied with such deductions as are clear and unimpeachable, and may serve to render comparison more easy.

TABLE showing the proportion of Deaths at different Ages, out of 10,000 Deaths of Males, of Females, and of both sexes, according to the Registers of Burials 1813—1830, and according to the Registers of Deaths for the year ending June 30, 1838.

| Age. | Registers of Burials. | | | Registers of Deaths. | | |
|---|---|---|---|---|---|---|
| | Males. | Females. | Both Sexes. | Males. | Females. | Both Sexes. |
| Under 1 year | 2,188 | 1,756 | 1,975 | 2,339 | 1,933 | 2,140 |
| 1 to 4 | 1,498 | 1,450 | 1,474 | 1,742 | 1,780 | 1,760 |
| 5 " 9 | 437 | 410 | 424 | 457 | 462 | 460 |
| 10 " 19 | 579 | 636 | 607 | 554 | 647 | 599 |
| 20 " 29 | 724 | 839 | 781 | 738 | 833 | 785 |
| 30 " 39 | 621 | 725 | 672 | 655 | 715 | 683 |
| 40 " 49 | 649 | 670 | 659 | 653 | 632 | 643 |
| 50 " 59 | 715 | 784 | 700 | 656 | 621 | 638 |
| 60 " 69 | 911 | 922 | 917 | 813 | 823 | 818 |
| 70 " 79 | 1,012 | 1,086 | 1,049 | 830 | 883 | 856 |
| 80 " 89 | 584 | 700 | 641 | 473 | 556 | 514 |
| 90 & upwards | 75 | 116 | 95 | 94 | 115 | 104 |

The largest and most important collection of facts relative to deaths at various ages made hitherto in this country, is the Table given in the Preface to the Population Returns for 1831, containing the ages of 3,938,496 persons, (of whom 1,996,195 were males, 1,942,301 females,) buried in England and Wales during 18 years, 1813—1830. In the subjoined table, I have afforded the means of comparing the result of this Registration of Burials with those of the Registration of Deaths for the year ending June 30, 1838, reducing each to the same denomination, and exhibiting their respective proportions to a common total. The difference between them will be found to consist chiefly in the larger proportion of deaths of infants, which appear in the Registration of Deaths— a difference which was to be expected; for it was known that it was in the record of such deaths that the Registration of Burials was principally deficient.

*Longevity in different parts of the Kingdom.*

Among the diversities, says the Registrar, which especially demand attention, and by which there is least danger of being led to false conclusions, are those which relate to longevity, showing the varying proportions of deaths in old age in different portions of the kingdom. From a few instances of extreme longevity no inference can be safely drawn; but the fact that of the deaths in any district a comparatively large proportion is above the age of 70, is a strong presumption in favour of the health of that district. These proportions will be found to vary greatly. In the whole of England and Wales, out of 1000 deaths, 145 have been at the age of 70 and upwards; while in the North Riding and northern part of the West Riding of Yorkshire, and in Durham, except the mining districts, the proportion has been as high as 210. In Northumberland, (excluding the mining district,) Cumberland, Westmoreland, and the north of Lancashire, the proportion has been 198; in Norfolk and Suffolk 196; in Devonshire 192; and in Cornwall 188.

In contrast with this evidence of the large proportion of persons who attain to old age in these more thinly-peopled portions of the kingdom, we find results extremely different where the population is densely congregated. In the metropolis and its suburbs the proportion who have died at 70 and upwards has been only 104; and even this proportion is favourable when compared with that of other large towns—the proportion in Birmingham being 81; in Leeds 79; and in Liverpool and Manchester only about 63.

Every fact which comes out displays the comparative salubrity of rural districts and insalubrity of large towns. The Army Reports, the Registrar's, and the limited experience of private practice, severally combine to prove this. London too bears the palm from the great commercial cities of England, and it is not a little curious that Manchester, which was placed by Dr. Hawkins highest on the scale, figures here the lowest.

*Deaths at particular Ages.*

A very marked diversity also appears in the proportion of deaths of infants in different parts of the country. In the mining parts of Staffordshire, and Shropshire, in Leeds and its Suburbs, and in Cambridgeshire, Huntingdonshire, and the lowland parts of Lincolnshire, the deaths of infants under one year have been more than 270 out of 1000 deaths at all ages; while in the northern counties of England, in Wiltshire, Dorsetshire and Devonshire, in Herefordshire and Monmouthshire, and in Wales, the deaths at that age out of 1000 at all ages scarcely exceeded 180.

The Registrar adds some very important observations:—

"But it is less essential that I should point out diversities which examination of the appended table will easily detect, than that I should advert to other circumstances which must also be borne in mind by those who would avoid erroneous inferences. The comparative table alone will not accurately indicate the comparative list of deaths at different ages, in different divisions of the kingdom, unless it be true that in each division there is the same proportion of persons living of the same age. But such an assumption is utterly disproved by the enumeration of the ages of persons living in May, 1821, made under the authority of the Population Act, which shows a wide difference in the proportions which were then found in the several counties.

To illustrate the risk of error from inattention to that circumstance, I will compare the deaths between the ages of 20 and 50 in Division 1, comprising the metropolis, with those in Division 6, comprising Hertfordshire, Bedfordshire, and Buckinghamshire, and the rural parts of Middlesex. It appears that in Division 1, the deaths between those ages out of 1000 at all ages are 241, while, in Division 6, they are only 192; from whence arises apparently an undeniable inference that there is some cause operating in the metropolis tending to cut short life when it has run through about half its natural course, which does not equally affect it in the adjacent rural districts. But it appears from the enumeration made in May, 1821, that of 10,000 persons of both sexes then living in Middlesex, there were 4,522 between the ages of 20 and 50; while the mean number of persons of the same age in Bedfordshire, Buckinghamshire, and Hertfordshire was only 3,581, a remarkable difference, attributable to the circumstance of persons born in the counties near London quitting them, as they emerge from childhood, to seek employment in the capital. There were thus 4,522 persons exposed to the risk of death, between the ages of 20 and 50, in Middlesex, for 3,581 in those three adjacent counties, and if the mortality were equal, the deaths between those ages in Middlesex and in the other three counties ought to bear the same proportion. Therefore, upon the supposition that the proportions of the living remain the same as in 1821, the results exhibited by the Abstract of Deaths will not be unfavourable to the metropolis.

Again, on comparing the proportion of deaths under 5 years of age in Division 19, (comprising Lancashire, south of Morecambe Bay, with the exception of Manchester and Liverpool,) with the same in Division 25, comprising Herefordshire, Monmouthshire, and Wales, it appears that in the former division there were 458 deaths, and in the latter 365. But on referring to the table, founded on the enumeration of 1821, it appears that out of 10,000 persons of each sex in the county of Lancaster exposed to the risk of death, under the age of 5, there were 1,711 males, and 1,582 females; while in Hereford the proportion was only 1,317 males, and 1,326 females; in Monmouthshire, 1,320 males, 1,404 females; in Wales (collectively) 1,514 males, 1,382 females. If, therefore, the proportion of the living at the above-mentioned age continued to be the same in the year ending June, 1838, and the mortality at that early age were the same in both Divisions, the proportion of deaths in Division 19 would of necessity be greater than in Division 25.

Being convinced that a consideration of these facts is essential as a corrective to undue inferences which might otherwise be drawn from the Abstract of Deaths I have caused the above-mentioned table, which may be found among the preliminary observations to the Population Abstracts of 1821, with the addition of the same for Wales collectively, to be appended to the Abstracts of Deaths. It is possible that the proportions there set down may have varied greatly in the lapse of 18 years; they are, therefore, re-produced for reference, not as affording sure grounds for calculation, but as warnings and correctives to the erroneous calculations which might otherwise be made. If, at the next ensuing census, a similar enumeration shall be made successfully, its results, combined with the registered deaths of an equal number of antecedent and succeeding years, will afford to the public the most important and useful materials for the solution of all questions depending on the duration of human life that have ever yet been possessed." 25.

The bulk of the volume is made up of circulars, abstracts, and tables, which it is impossible for us to notice. They are of great value as statistical documents, and will, we do not doubt, be amply consulted. But there is a circular from the Registrar, and a letter to him, which should not be so dismissed.

### Circular and Appended Exlanatory Statement addressed to Medical Practitioners, respecting the Registration of the Causes of Death.

This Circular emanates from the Colleges of Physicians and Surgeons, and from the Society of Apothecaries, and is addressed to the profession. It appeared at the time in the medical journals, but a year having passed, the system being in full operation, and the advantages which must accrue to science from its success being so striking, we think it right to re-circulate the Circular, and to lean on its recommendations with all the weight that we possess. We would intreat our readers to contribute to their utmost, towards rendering the Registration of the Causes of Death a perfect one. By doing so they will be rendering a most important service both to society and science. We now quote the Circular.

The recent Act for registering Births, Deaths, and Marriages in England presents an opportunity for obtaining that great *desideratum* in medical statistics, a more exact statement of *the causes of death*, in the case of every registered death throughout the whole of England and Wales, after the month of June next ensuing.

The Register-Books in which all deaths are to be registered after the last day of June, 1837, contain columns wherein may be inserted the *cause of death*, in juxtaposition with those other important illustrative circumstances, the *sex*, the *age*, and the *profession* or calling of the deceased person. Each Register Book will also be assigned to a particular District of small extent, and will thus show in what part of the kingdom each death has occurred. If, therefore, the cause of death be correctly inserted, there will exist thence-forward public documents, from whence may be derived a more accurate knowledge, not only of the comparative prevalence of various mortal diseases, as regards the whole of England and Wales, but also of the *localities* in which they respectively prevail, and the *sex*, *age* and *condition of life*, which each principally affects.

For the attainment of this object, it is necessary to ensure, as far as it is possible, the correct insertion of the "*cause of death.*" It is obvious that on this subject the requisite information can seldom be given to the Registrar, except by the medical attendant of the deceased person, and that even if the Registrar be a medical practitioner (which in many instances will be the case), yet will he often be unable to ascertain the truth in this respect, if he is to depend solely on the reports of persons ignorant of medicine, and of the names and nature of diseases; and it cannot be expected that from his own knowledge he will be able so far to correct their errors, as to ensure a statement worthy of credit. The requisite information must therefore be supplied either *directly* or *indirectly* by the medical attendant of the deceased person;—that is to say, if such medical attendant is not applied to by the Registrar, he must afford the requisite information to those other persons to whom the Registrar must apply.

The persons who according to the Act for Registering Births, Deaths, and Marriages in England, must give information to the Registrar on being requested so to do, are "some person *present at the death*, or *in attendance* during the last illness," or "in case of the death, illness, inability, or default of all such persons, the *occupier* of the house or tenement, or, if the occupier be the person who shall have died, some *inmate* of the house or tenement in which such death shall have happened." It is also provided that, "for the purposes of this Act, the master or keeper of every gaol, prison, or house of correction, or workhouse, hospital, or lunatic asylum, or public or charitable institution, shall be deemed the *occupier* thereof."

It is therefore earnestly recommended that every practising member of any branch of the medical profession who may have been present at the death, or in attendance during the last illness of any person, shall, immediately after such death, place in the hands of such other persons as were in attendance, of the occupier of the house in which the death occurred, and of some inmate who may probably be required to give information, *written statements of the cause of death*, which such persons may show to the Registrar, and give as their information on that subject.

It is desirable that such statement should be very short, the column in the Register Book in which it is to be inserted being not more than sufficient for the insertion of about ten words of moderate length. It should therefore contain only the name of the disease which was considered to be the cause of death, and not a *detailed* account either of antecedent symptoms or of the appearances which may have presented themselves after death. It is also desirable that such statement should exhibit the popular or common name of the disease, in preference to such as is known only to medical men, whenever the popular name will denote the cause of death with sufficient precision.

One of the appendices to this Report consists of a letter to the Registrar General from Mr. William Farr, a gentleman known to all our readers for his indefatigable exertions in promoting medical statistics.

This letter presents abstracts of the recorded causes of 141,607 deaths, which were registered in England and Wales during the half-year ending December 31st, 1837. With the following observations we cordially concur.

"One of the many obvious applications of the facts will be to the promotion of practical medicine. The extent to which epidemics vary in different localities, seasons, and classes of society, will be indicated by the registered diseases; and the experienced practitioner, wherever he may be placed, will learn to administer remedies with discrimination, and with due reference to the circumstances of the population. He will discover that the characters of diseases change, and will not treat a pneumonia in the same way in Whitechapel and in Westmoreland, if it appear, from the causes of death, that the diseases and constitution of the population present striking discrepancies. The modifications in the character of diseases, and in the medical treatment, are indicated perhaps more accurately by the prevailing epidemics than by either the temperature, the hygrometricity, or any other appreciable condition of the atmosphere; and it was Sydenham's doctrine, that the treatment of acute diseases should have a reference not only to the immediate symptoms, and to the seasons, but also to the epidemic constitutions of the year and place. A national system of registration like the present can alone indicate the characters of the diseases in every district; and determine how far the practice taught in the schools, or illustrated in crowded hospitals, and in the unhealthy parts of the metropolis, among the destitute poor, may require modification among other classes of society, and in other localities.

The registration of the diseases of the several districts will furnish medical men with a series of valuable remedial agents. It will designate the localities where disease is most rife, and where there is the least tendency to particular classes of sickness and infirmity. In recommending a residence to patients the physician will find the registered causes of death an indispensable directory; and the utility of a sanatory map of the country, such as the returns will furnish, cannot fail to be felt in England, where a part of the population is constantly migrating from place to place in search of health. Much information has been collected respecting the influence of the English climate; but the facts will bring to light many salubrious spots hitherto unknown, and disclose the dangers which infest others unsuspected. Invalids resort to some unhealthy places; families carry their children in the Autumn into districts where small-pox and measles are often epidemical, or go into parts of the country where, as the registration shows, bowel complaints and fevers are extraordinarily fatal.

The registration of the causes of death, besides contributing to practical medicine, will give greater precision to the principles of physic. Medicine, like the other natural sciences, is beginning to abandon vague conjecture where facts can be accurately determined by observation; and to substitute numerical expressions for uncertain assertions. The advantages of this change are evident. The prevalence of a disease, for instance, is expressed by the deaths in a given time out of a given number living with as much accuracy as the temperature is indicated by a thermometer; so that when the mean population of the district is known, the rise and decline of epidemics may be traced exactly, and it will then be possible to solve the problem, whether certain tribes of epidemic disorders constantly follow others, in one determined series or cycle. Loose phrases are still current, for which numerical formulæ will be substituted. Sydenham, one of the most accurate of medical writers, in speaking of small-pox, employed such terms as these: (1661) 'It prevailed a little, but disappeared again.' (1667-9) 'The small-pox was more prevalent in town for the first two years of this constitution than I ever remember it to have been.'—(1670-2.) 'The small-pox arose; yielded to the dysentery; returned,' &c. &c. These terms admit of no strict comparison with each other; for it is difficult to say in which year the small-pox was most fatal, and impossible to compare Sydenham's experience thus expressed with the experience of other writers in other places and other ages; for 'prevailed a little,' 'raged with violence,' and similar terms may imply either that small-pox destroyed 1, or 2, or 5, or 10 per cent. of the population." 87.

It cannot be forgotten how egregious a fallacy, how monstrous an absurdity was promulgated in a controversy upon erysipelas, a few years ago by an eminent hospital surgeon. Where, said Mr. Lawrence, does the region for bark and that for bleeding begin? At what particular mile-stone from the city does disease alter its character? This logical quibble was directed against the idea of an essential difference of the medical constitution of the country and the town—an idea dictated at once by reason, sanctioned by individual experience, and confirmed in the most positive manner by the registry.

Mr. Farr adds:—Only a limited number of facts fall under the notice of a single observer. His opinions, when they are the results of his own experience, are stated in general terms, and are often adopted by others in entirely different circumstances. Notwithstanding the constancy of Nature, this leads to serious practical errors. Hippocrates wrote his immortal works in Asia Minor and Greece, in a particular climate, stage of culture, and civilization; yet all his precepts were taken for the guide of his successors in England, France, and Germany. The therapeutic doctrines of Sydenham, who lived in Pall Mall, and practised principally in Westminster, spread through Europe. The celebrated Broussais' theory of irritation and *gastro-enterite* originated in the French camps. The physicians of this country, when the causes of death are universally recorded, and recorded accurately, will be saved from the fallacies of partial generalization; and, with the results of the registry before them, will be enabled to obtain extended views of the nature, courses, and modifications of diseases. They will have, as a basis for deduction, their own observations, and those of every medical practitioner in England and Wales, in all places, and in all times; for the national registration goes on in Winter and Summer, in Spring and Autumn; and it extends to all parts of the kingdom and to all classes of society.

The works of Huxham, Haygarth, Short, Heysham, Heberden, Willan, Bateman, and many recent medical writers, present illustrations of all these propositions; although they rarely had access to sources more authentic than the Bills of Mortality.

Any improvement in the treatment of disease, and any addition to medical science, will tend ultimately to the diminution of human suffering; but the registration of the causes of death is calculated to exercise a still more direct influence upon public health. Diseases are more easily prevented than cured, and the first step to their prevention is the discovery of their exciting causes. The registry will show the agency of these causes by numerical facts, and measure the intensity of their influence. The annual rate of mortality in some districts will be found to be 4 per cent., in others 2 per cent.; in other words, the people in one set of circumstances live 50 years, while in another set of circumstances, which the registration will indicate, they do not live more than 25 years. In these wretched districts, nearly 8 per cent. are constantly sick, and the energy of the whole population is withered to the roots.

Mr. Farr believes that, by proper hygienic measures, it is possible to reduce the annual deaths in England and Wales by 30,000, and to increase the vigour of the population in an equal proportion. Of this we ourselves entertain no doubt. When we look to the obvious causes of disease which are abounding—to the intemperate habits—the immoral licence—the crowded habitations—and the filthy quarters of the poor in towns, we cannot but feel how materially a diminution, not to say suppression, of these evils must contribute to the health, the energy, and the happiness of the English people. Growing as the population is in numbers, agitated as it is with the inquiry into the nature of political institutions, it becomes yearly a more anxious subject of solicitude to Government, and of interest to humane and thinking men. Some of the difficulty of bettering the people's condition will assuredly be lightened by medicine and statistics.

We appeal to every member of the profession to contribute, so far as he has the power of contributing, to the accumulation of *genuine* facts. The

*accuracy* of the Register is every thing. If the causes of death are set down in a slipslop fashion, if vague, and gossiping, or inaccurate returns are made, science will be poisoned at its roots, and the error will be the more fatal and more gross, because an error of numbers.

Mr. Farr makes some observations on *Statistical Nosology*, for which we regret that we have not room. We think it would be well, if the names of diseases were better agreed on, but we think it is of even more importance to give *the* correct designation, however circumlocutory. Mr. Farr suggests that:—

In order to render the Register as correct as possible, it is desirable that the cause of death should be directly certified in every instance by the medical attendant, who might either leave the certificate with the informant, or give it upon application to the registrar. When the medical attendant is the informant, he will of course sign the register, as directed by the Act. The certificates of the cause of death might be in one of the subjoined forms, which present examples of some of the more complicated cases. The duration of the fatal disease should be stated, *when known*, in hours, days, or years, which should supersede the words "sudden," &c., and in the end furnish many highly important results. The registrar should insert the terms corresponding to those in italics in the column of the register headed "Cause of Death."

Edward Davies, aged 11, died of

*Typhus, terminating in pneumonia, after* 15 *days' illness.**

(Signed)

* The primary and secondary diseases should be specified in the Registers.

Thomas Williams, aged 70, died of

*Apoplexy (second attack, of* 1 *day's duration), with effusion of blood into the ventricles of the brain.*

(Signed)

Mary White, aged 40, died of

*Carcinoma of the breast, of* 2 *years' continuance.**

(Signed)

* The *nature* of the disease, and the *parts* affected should be specified in cases of this kind.

Mr. Farr appends a great number of Tables, and presents us with a running commentary upon them. It would be impracticable to present the Tables themselves, or even to offer a continuous line of commentary. We must content ourselves with picking out such passages and facts as appear to us instructive.

*Mortality from Influenza in* 1837.—Influenza prevailed epidemically in the beginning of the year 1837, and destroyed great numbers. It subsided in March, and the rest of the year was considered healthy. The number of burials collected and published by the London parish-clerks in the months of

January and February (or rather 20th December 1836, to 21st February 1837), were 2,053 and 2,336, while the average monthly burials of the next nine months were 1,355.*

These wholesale omissions of the Parish Clerks are certainly rather startling.

*Mortality per Cent in the last half year of* 1837.—Calculated from the registered deaths in England and Wales, for the last half year of 1837, the annual rate of mortality per cent. was then found to be nearly:—

| | Males. | Females. | Mean of the two Sexes. | |
|---|---|---|---|---|
| | 2·08 . . | 1·97 . . | 2·02 | per cent. |
| or, | 1 in 48 . . | 1 in 51 . . | 1 in 49 | |

*Deaths from Small-pox.*—Small-pox destroyed 5,811 lives in the half year. It will be seen subsequently that small pox was epidemic in several parts of the country, particularly in Liverpool, Bath, and Exeter. The following were the ages at which 1,056 of the deaths occurred in Bath, Liverpool, Exeter, parts of Shropshire, Worcestershire, and the Metropolis.

| Age .... | 0—4 | 5—9 | 10—14 | 15—19 | 20—29 | 30—39 | 40—49 | 50—59 | All ages. |
|---|---|---|---|---|---|---|---|---|---|
| Deaths. | 887 | 99 | 15 | 18 | 29 | 5 | 2 | 1 | 1,056 |

This specification of the ages may be considered an approximation to the ages of the 5,811. It has a direct bearing on a very important practical question which has recently engaged attention, namely, whether the prevalence of small-pox is due to the diminished influence of vaccination, or whether the protective power of vaccination progressively declines, so that a child vaccinated at 2 years of age is more susceptible of small-pox at the age of 20, or 30, than at any earlier age. The facts in the registers would decide the question if the medical attendant ascertained whether the individuals who died of small-pox had ever been vaccinated, and if this fact and the interval between vaccination and death were entered: which might easily be done. In the mean time it seems exceedingly probable that the majority of the 5,811 had never been vaccinated, as they were very young, and when the poorer classes do not neglect vaccination altogether, they often defer it for

* It may not be uninteresting to compare the results of the registration with the London bills of mortality. The total burials in the Bills of 1837 amounted to 21,063; and in the six months 21st June to 26th December 1837, corresponding nearly with the half year during which the Registration Act had been in operation, the number of burials in the Bills was 10,518 ("Gentleman's Magazine," 1837). The number of deaths registered in the same parishes under the Act was 18,266; and as 4,450 burials, many of which occurred in the first half of the year, are set down to December, it may be safely asserted that the parish clerks registered little more than half the deaths that occurred within the limits of the London bills of mortality.

years. Vaccination is delayed too long by all classes; it should not be practised later than the first three months, as the early deaths at Bath and Liverpool testify.

*Deaths from Mental Emotions.*—Ten deaths—nine of females, one of a male—are ascribed to mental emotions of one kind or other; seven to fright, one to grief for the death of a son, and two to a broken heart. It is well established that grief and distress are the roots of various organic diseases, and many cases are recorded in which sudden mental shocks have destroyed life, or induced madness. Where life is destroyed instantly, the connexion of the shock with the death is less equivocal than in cases where a considerable interval elapses between the two events. It happens that in two of the cases the alleged cause, as well as the deaths, were recorded. A female, aged 63, it is said, died November 7th from trouble for the death of her son; and from another page of the register it appears that the son's death occurred October 30th. Again the death of a female, aged 41, on November 14th, is ascribed to "fright," occasioned by the sudden death of her brother; and her brother died on the 19th of October.

*Deaths from Consumption.*—The deaths from this dreadful malady amounted to 27,754 = 20 per cent. of the total number of deaths; or nearly 4 annually, out of 1000 living.

*Diseases of the Digestive Organs.*—5,115 males and 4,735 females died of disease of the digestive organs, and the annual rate of mortality in the males was 1·5, in the females 1·3, out of 1000 living. Nearly all diseases of the intestinal canal proved most fatal to males, so did jaundice and all the diseases of the liver, except hepatitis. Hernia is much more common in males than females, yet the mortality in the two sexes—·044 and ·029 per 1,000—did not differ so widely, and the reason of this is, that the bowel when it does escape is much more liable to strangulation in the hernia of females than in the hernia of males.

*Diseases of the Urinary Organs.*—Diseases of the urinary organs destroy five times as many males as females,—the rate of mortality of the two sexes, under this head, having been ·199 and ·037 per 1,000. The disparity has been ascribed to mechanical causes; but will a mechanical explanation account for the fact that 68 males and only 27 females died of diabetes? Dr. Yelloly, in a paper published in the Philosophical Transactions, estimated that 1 in 108,000 persons was cut annually for stone in England and Wales. It appears from the table that 47 in 1,000,000 males, and 5 in 1,000,000 females, die of stone and gravel. The latter, it must be admitted, is a vague term in popular language; but the mortality from stone is certainly 1 in 100,000 annually. Bright's disease is registered "Disease of the Kidneys." The coagulability of the urine is often undetected by careless practitioners. A female child, aged two years, is stated to have died *suddenly* of diabetes.

*Diseases of the Tegumentary System.*—The mortality from all the diseases of the skin *and* of the digestive organs is about 1,976 per 100, and it is a curious coincidence that the mortality from all the diseases of the digestive organs is nearly the same—2·067. The diseases of the connected internal and external membranes are equally destructive; but the mortality from diseases of the two systems together is only 4·, while that from diseases of the respiratory organs is 5·5 per 1,000.

*Intemperance and Starvation.*—Males are, according to the registers, more intemperate than females, as the deaths of 70 males and of 15 females were ascribed directly to intemperance; of 67 males and 12 females to gout; of 86 males and 9 females to delirium tremens (in the second class). It will be seen with regret that in the half-year the deaths of 63 individuals were ascribed (principally at inquests) to starvation; this is almost 1 annually to a population of 111,000. The want of food implies the want of everything else—except water—as firing, clothing, every convenience, every necessary of life, is abandoned at the imperious bidding of hunger. Hunger destroys a much higher proportion than is indicated by the registers in this and in every other country; but its effects, like the effects of excess, are generally manifested indirectly, in the production of diseases of various kinds. The privation is rarely ever absolute; the supply of food is inadequate to supply the wants of the organization, which requires daily animal or vegetable matter containing not less than nine ounces of carbon.

*Ages of Forty-four Suicides.*

| Age ...... | 10—20 | 20—30 | 30—40 | 40—50 | 50—60 | 60—70 | 70—80 | All ages. |
|---|---|---|---|---|---|---|---|---|
| Males..... | 1 | 5 | .. | 3 | 4 | 1 | .. | 14 |
| Females .. | 5 | 7 | 2 | 3 | 8 | 3 | 2 | 30 |
| Both Sexes. | 6 | 12 | 2 | 6 | 12 | 4 | 2 | 44 |

*Diseases of Towns and of the Country.*

Mr. Farr devotes several tables and many observations to this most important subject. It comes home individually to all of us—it affects the great question of the Poor Law—and it is a matter of the utmost consequence to the Government of the country. The means of improving, physically as well as morally, the lower classes of the town population, must speedily become a source of anxious consideration to the Legislature. The real state of the case, and the actual nature of the evil must be understood before any remedy can be applied, and statistical tables will give us the requisite information.

The area, says Mr. Farr, of England and Wales is 57,805 square miles; and as the land populatiton in the period under investigation was about 15,330,000, the number of inhabitants to a square mile was 265. The population is very unequally distributed; being thinly scattered over the agricultural districts, and accumulated at different intervals in villages, towns, and cities, where, as for instance in the metropolis, in the Unions of St. Giles and St. George Bloomsbury, the Strand, the City of London, East and West London, Holborn, St. George in the East, and St. Mary Whitechapel, the number of inhabitants to a square mile is 123,904. The population increased very slightly in these districts in the interval between the censuses of 1821 and of 1831; whence it may be inferred that the ground is nearly all occupied. In the East and West London Unions, however, the population is still more dense; the number of inhabitants to a square mile is 186,046. The greatest density attained in the heart of English cities is therefore nearly 243,000 inhabitants to a geographical square mile.

We imagine that some mistake has been made in the latter computation. Be that as it may, Mr. Farr gives a table, which exhibits two sets of observations—the deaths in the metropolitan division, with a population of 1,790,451, lodged upon an area of 70 square miles, and the deaths and fatal diseases in Devonshire, Dorsetshire, Wiltshire, Cornwall, and Somersetshire, where nearly the same number of inhabitants (1,723,770) was distributed over an area of 7,933 square miles. The five counties form the south-western division of England, and are bounded by the sea and a line running from Studland Bay to the Avon.

It appears that the area of square miles in the metropolis is 70—in the counties, 7,933. The estimated population, 1st of October, 1837, was, in the metropolis, 1,790,451,—in the counties, 1,723,770. The population to a square mile, in the metropolis, 25,578—in the counties 222. Let us look at the mortality.

| | Metropolis. | Five Counties. |
|---|---|---|
| Deaths, 1st July to 31st December 1837 | 24,959 | 15,210 |
| ,, 1st January to 30th June 1838 | 28,638 | 18,864 |
| | 53,597 | 34,074 |

In comparing the deaths from different diseases in the metropolis with those in the counties it will be recollected that the counties include Exeter, Plymouth, Portsmouth and Portsea, Southampton, Bath, and a great number of towns; that small-pox was epidemic in Bath and Exeter; dysentery in Taunton; and that the health of this half-year in the entire extent of country was by no means favourable.

We are not clear how Portsmouth, Portsea, and Southampton, can be taken out of Hampshire, and given to the "five counties."

Mr. Farr proceeds to examine the particular classes of disease which are rife in country and town. It appears that the crowding of cities doubles the mortality from epidemic, endemic, and contagious diseases, as well as from diseases of the nervous system—the ratio of deaths having been as 1 to 2·11, and 1 to 2·13; and upon reference to the individual diseases in Tables C. D. it will be seen that the augmentation in the latter class occurs principally in convulsions and hydrocephalus:—Deaths by convulsions, counties, 1,347, cities, 3,723, ratio 1 : 2·76; by hydrocephalus, counties 559, cities 1,540, ratio 1 : 2·75. It has already been intimated that convulsion is a frequent intercurrent symptom in diarrhœa and diseases of the epidemic class in infants; it may exist, however, as an independent affection, and in that case has clearly, as well as hydrocephalus, with which it is allied, an epidemic character. A similar remark will apply to pneumonia and bronchitis, of which 1,209 cases were registered in the counties, 2,865 in the cities; ratio 1 : 2·37. The pulmonary inflammation was, in many cases, developed in the course of measles, influenza, and other diseases of the first class. The three following diseases, which principally affect adults between the ages of 15 and 65, show that unhealthy places augment the fatality of diseases in different degrees.

| | Counties. | Cities. | Increase per cent. in Cities. |
|---|---|---|---|
| Deaths by consumption | 5,857 | 8,125 | 39 |
| " childbirth | 217 | 372 | 71 |
| " typhus | 1,564 | 3,456 | 221 |

This gives the classification a peculiar property. Wherever the absolute mortality is low, the number of deaths in the epidemic class is less than the number in the pulmonary class; and, on the contrary, wherever the deaths in the first class exceed or equal those in the third, it may be affirmed that the absolute mortality is high.

"The occupations in cities," remarks Mr. Farr, "are not more laborious than agriculture, and the great mass of the town population have constant exercise and employment; their wages are higher, their dwellings as good, their clothing as warm, and their food certainly as substantial as that of the agricultural labourer. The Poor Law Inquiry, and successive Parliamentary Committees, have shown that the families of agricultural labourers subsist upon a minimum of animal food, and an inadequate supply of bread and potatoes. The source of the higher mortality in cities is, therefore, in the insalubrity of the atmosphere." 111.

"All gases and effluvia, like odours, are diffusible; they have a certain force of diffusion, which Professor Graham has expressed numerically; and all the emanations from human habitations in the open country mingle, almost as soon as they escape, in the currents of the atmosphere. But locate, instead of one individual to a square mile of land (the supposed density of population in the uncultivated forests of America and the steppes of Asia,) 200,000 individuals upon a square mile, as soldiers in a camp, and the poison will be concentrated 200,000 fold; intersect the space in every direction by 10,000 high walls, which overhang the narrow streets, shut out the sunlight, and intercept the movements of the atmosphere; let the rejected vegetables, the offal of slaughtered animals, the filth produced in every way decay in the houses and courts, or stagnate in the wet streets; bury the dead in the midst of the living; and the atmosphere will be an active poison, which will destroy, as it did in London formerly, and as it does in Constantinople now, 5-7 per cent. of the inhabitants annually, and generate, when the temperature is high, recurring plagues, in which a fourth part of the entire population will perish. But the health will be little more impaired by residence upon one than upon 100 square miles, if means can be devised for supplying the 200,000 individuals with 200,000,000 cubic feet of pure air daily, and for removing the principal sources of poisonous exhalations. The latter object is partly accomplished by paved, even streets, by the scavenger, by an abundant supply of water, by large well-constructed, trapped sewers, and by domestic habits of cleanliness: but it is difficult to perceive how volatile impurities can be removed, and how a stream of uncontaminated air can be supplied where the sun cannot heat the earth and air, where there are no open squares, or the streets are narrow, or the houses are only separated by courts, or built in *cul de sac*." 112.

The fact of the greater insalubrity of cities, suggesting itself, as it has ever done, to the common observation and common sense of men, is now stamped by the authority of statistics, and acquires a degree of precision and specification which must ultimately prove highly useful. The next point is to take the country or the town separately, and ascertain the particular spots most healthy or the contrary. This being done, and the circumstances of those spots being known, we arrive at the causes of disease, and are directed towards its prevention.

Mr. Farr takes thirty-two metropolitan districts, and examines the mean annual mortality of females from the 1st July to 31st December 1837, excluding the deaths in hospitals. It appears that, in Whitechapel, the mortality is highest, the annual deaths per cent. being 3·908; while, in St. George, Hanover-square, it is lowest, the deaths being only 1·785 per cent. But it is singular (and, to our minds, carries the conviction of the existence of some fallacy) that the city of London is almost as healthy as St. George, Hanover-square, being numbered at 1·980; while Kensington, healthy as it is generally thought, is set down at 2·190. Can the anomaly depend on any arrangement in the City for disposing of its poor, or in the country residences of its denizens? We throw this out as a hint, for we feel staggered by the seeming anomaly.

It will be found, says Mr. Farr, *cæteris paribus*, that the mortality increases as the density of the population increases; and where the density and the affluence are the same, that the rate of mortality depends upon the efficiency of the ventilation, and of the means which are employed for the removal of impurities.

We quote the following brief table, and the remarks which follow it.

TABLE H.

Exhibiting the MEAN MORTALITY, in Three Groups, of the Thirty-two Metropolitan Districts.

| Districts. | Square Yards to One Person. | Annual Rate of Mortality per 100. | Annual Rate of Mortality per Cent. by Diseases of | | | | | | |
|---|---|---|---|---|---|---|---|---|---|
| | | | The Epidemic Class. | *Typhus.* | The Nervous System. | The Respiratory System. | *Phthisis.* | The Digestive Organs. | Other Classes. |
| 1 to 10, (mean) | 57 | 3·321 | ·991 | ·324 | ·543 | ·822 | ·478 | ·208 | ·758 |
| 11 to 20, ,, | 78 | 2·839 | ·701 | ·205 | ·467 | ·768 | ·451 | ·197 | ·706 |
| 21 to 30, ,, | 217 | 2·163 | ·485 | ·107 | ·369 | ·588 | ·354 | ·155 | ·567 |

The mortality then increases with the density, yet the densest districts are not invariably the most unhealthy.

| Unions or District. | Area in square yards to one person. | Annual rate of Mortality per cent. |
|---|---|---|
| St. James, City of London, Strand (mean) | 24 | 2.1 |
| Shoreditch, Bethnal Green, Bermondsey (mean) | 60 | 3.1 |

The necessary deduction from the double series of facts, then, is that the mortality has a tendency to increase as the density of the population increases, but that the unhealthful tendency can be counteracted by artificial agencies. In other terms, the mortality of cities in England and Wales is high, but it may be immeasurably reduced. A good, general system of sewers; the intersection of the dense, crowded districts of the metropolis by a few spacious streets; and a park in the East end of London would probably diminish the annual deaths by several thousands, prevent many years of sickness, and add several years to the lives of the entire population. Similar improvements would have the same effects in the other cities of the empire. The poorer classes would be benefitted by these measures, and the poor-rates would be reduced; but all classes of the community are directly interested in their adoption, for the epidemics, whether influenza, typhus, or cholera,—small-pox, scarlatina, or measles, which arise in the East end of the town, do not stay there; they travel to the West end, and prove fatal in wide streets and squares. The registers show this; they trace diseases from unhealthy to healthy quarters, and follow them from the centres of cities to the surrounding villages and remote dwellings." 116.

The rich, in this country, will only look after the poor, when they find it their interest to do so. That case seems to be pretty well made out already. The poor are costing the other classes a smart sum in purse and person, and what with troops, constabulary, fever-hospitals, and fevers, the account is running up pretty handsomely. So the legislature will try its hand at a nostrum anon.

In the unhealthy districts of the metropolis, the mortality from the epidemic class of diseases is higher than the mortality from diseases of the respiratory system; in the healthy districts, on the contrary, the mortality by the former is lower than the mortality by the latter class of diseases.

*Annual Rate of Mortality per Cent.*

| Districts. | From the Epidemic class of Diseases. | From Diseases of the Respiratory Organs. | Absolute Mortality from all causes. |
|---|---|---|---|
| 1—10 (unhealthy).. | ·99 | ·82 | 3·32 |
| 11—20 .......... | ·70 | ·77 | 2·84 |
| 21—30 (healthy) .. | ·48 | ·59 | 2·16 |

The reason of this is obvious: diseases of the epidemic class increase more rapidly than diseases of the respiratory system in unhealthy localities.

Mr. Farr, after offering some further tabular data, remarks that it may be laid down as a general principle that wherever the proportion of deaths from phthisis, compared with the total deaths, is high, the absolute mortality is low, and that the absolute mortality from phthisis itself is low. Attention to this fact will obviate several practical errors, such, for instance, as sending consumptive patients to the West Indies. The deaths out of the living express the real tendency to phthisis.

| | Districts 1—10. | Districts 11—20. | Districts 21—30. |
|---|---|---|---|
| Proportion of deaths by phthisis in 100 *deaths.* | 14·4 | 15·8 | 16·4 |
| Annual deaths by phthisis out of 100 *living* .... | ·478 | ·451 | ·354 |

Such are the facts in this Report to which we have thought it right to direct our readers' attention. They are fraught, we conceive, with a high interest. Yet they are only the "beginning of the end." We may expect much more that is valuable from the same quarter. We trust that the Registrar General will deem the furtherance of medical science one great object of his office, and we are sure that the profession will cordially express its sense of the zeal and the intelligence of Mr. Farr.

### II. SICKNESS AND MORTALITY OF TROOPS IN BRITISH AMERICA.

Our readers will remember that, in our last Number, we devoted a large amount of space to the medical returns from our troops in the Mediterranean. We postponed the examination of the Reports from British America, and to them we now beg to direct attention. We believe there can be but one opinion on the value of Reports of this description. To their influence medicine will assuredly be indebted for the correction of some errors and the establishment of some truths. Already it has been proved how erroneous is the idea, how pernicious the practice of sending invalids with pulmonary affections to the Mediterranean. It will probably come out that such patients receive as much injury as benefit from transplantation to any warm climate.

The stations occupied by our troops on the Continent and Coast of North America form four Military Commands:—

I. The Bermudas,
II. Nova Scotia and New Brunswick, including Cape Breton and Prince Edward's Island,
III. Upper and Lower Canada,
IV. Newfoundland.

### 1. *The Bermudas.*

These islands lie about 600 miles to the east of South Carolina, in latitude 32° 25′ N., longitude 64° 50′ W., and are said to exceed 300 in number, but most of them are barren rocks; four only are of sufficient importance to be garrisoned by troops, viz., St. George's, the Main, Somerset, and Ireland Island, and to these the Reporters confine themselves.

Compared with the West India Islands, the Bermudas are by no means elevated, the highest point not exceeding 200 feet. They are rocky and very destitute of soil, which in no part is more than two or three feet in depth, and generally consists of the debris of marine shells mixed with sand and vegetable mould. The substratum is a white porous rock, called "Bermuda stone," which is easily worked, but on exposure to the air becomes hard and of a blueish colour.

Though the surface is apparently so unfavourable to vegetation, a considerable part of these islands, particularly on the southern side, is covered with forests of lofty cedar.

In the larger islands are several lagoons or marshes, some of them communicating with the sea.

The climate of the Bermudas has been described by some authors as a perpetual summer. This description, however, appears to be rather overstrained; compared with the climate of northern regions, or even of the adjacent coast of America under the same latitude, it is certainly uniform; but the summer is exceedingly hot, even more so than in the West Indies, probably attributable to the light and scanty nature of the soil, which, being speedily divested of vegetation by the influence of solar heat and the absence of moisture, reflects the sun's rays from its arid surface with increased intensity. The absence of the regular trade winds, which serve to modify the heat in the West Indies, has by some been supposed another cause of the high range of temperature during the summer months, but this seems by no means probable in a climate where there is rarely a calm, and strong winds generally prevail from one quarter or other.

If the temperature of the summer months is compared with that of British Guiana, as shown in the West India Report, there will be found an excess of several degrees at this station, though 1500 miles further to the north.

We possess no exact measurement of the quantity of rain; the principal fall is between August and October, there are also very heavy showers in January and February, but seldom any during the summer months, and at that season dew is never deposited, though it is, occasionally, in winter. Southerly winds are most common during summer, and are said to be damp and oppressive; those from the north-east frequently blow with great violence during winter, and are dry, cold, and in many instances too keen for invalids. Winds from the west and north-west are most common in spring and autumn, and when from the latter point are generally accompanied by heavy falls of rain or thunder-storms.

The principal military station is at the town of St. George, situate on an island of the same name, about three miles in length, but in no part exceeding half a mile in breadth. It may be termed the capital of the Bermudas, and lies in a low confined situation near the eastern extremity of the island, at the foot of some high grounds which form a kind of amphitheatre around it, and, by impeding ventilation, add materially to the temperature.

From the table of Admissions into Hospital and Deaths, from 1817 to 1836 inclusive, it would appear that among every 1000 troops employed in this Command, 1310 cases of sickness have occurred in the course of the year, that is, every soldier has on an average, been under treatment for some disease or other once in nine months. But, in point of fact, the sickness has been greater, in consequence of a deficiency in the returns.

The deaths during these 20 years have averaged $32\frac{1}{3}$ per thousand of the strength annually, a very high ratio of mortality in a climate generally esteemed healthy; but a considerable part of this is attributable to the yellow fever of 1819, by which a fourth part of the force was cut off in a couple of months. Even deducting the extra mortality for that cause, however, the average of the other years considerably exceeds that of troops in Great Britain or the Mediterranean.

The principal forms of sickness have been diseases of the stomach and bowels, 415 per thousand annually—abscesses and ulcers, 191 per thousand—fevers, 136 per thousand—wounds and injuries, 135 per thousand—diseases of the lungs, 126 per thousand.

*Fevers.*—If the sickness and mortality by yellow fever in 1818 and 1819 be excluded, as that disease is comparatively of rare occurrence in these islands, the other types of fever will be found less frequent and less productive of mortality than at any of the Mediterranean stations; and it is especially worthy of remark, that notwithstanding the numerous marshy situations in different parts of this island, fevers of the intermittent type are almost altogether unknown: the only prevalent form is the common continued, which is observed in every climate and is seldom of a fatal character.

On the yellow fever we need say nothing, except that the usual difficulties are apparent in determining it's origin.

Eruptive fevers are exceedingly rare, only two cases having occurred among all the troops in the course of 20 years.

*Diseases of the Lungs.*—"Though this class of diseases, when taken in the aggregate, does not appear to be very prevalent in these islands, yet the most dangerous of them, viz., inflammation of the lungs and consumption, are decidedly so, and hence the above ratio of mortality is unusually high, being $8\frac{7}{10}$ per thousand of the strength annually, which is more than among troops in the United Kingdom, or any of the Mediterranean stations.

This appears more remarkable when we consider the uniformity of temperature at the Bermudas during a great part of the year, and the absence of those extremes of cold to which such diseases in northern latitudes are frequently attributed. It is sufficiently demonstrative of the erroneous nature of the ideas generally entertained on this head, particularly as regards consumption, that 9 per thousand of the troops at this station are attacked annually by that disease, of whom nearly three-fourths die before an opportunity offers for their removal, while in Great Britain the proportion attacked annually is but $6\frac{4}{10}$ per thousand, and we shall presently have occasion to observe, that even in the most inclement regions of British America the proportion is equally low.

Inflammation of the lungs, too, is exceedingly prevalent, without any perceptible cause in the climate to induce it; catarrhal affections and the other diseases of this class exhibit much the same degree of prevalence as among troops in the Mediterranean."

*Diseases of the Stomach and Bowels.*—On an average nearly one-half of the troops are attacked by diseases of the bowels annually, which is a larger proportion than even in the Windward and Leeward Command, though they are fortunately less severe in their character. This class of diseases is exceedingly prevalent here in every form, and in particular inflammation of the stomach, which elsewhere is of rare occurrence, has been so common, that in 1825 and 1826 a seventh part of the force was attacked by it. Their prevalence is attributed to the inadequate supply of fresh provisions.

*Delirium Tremens* is very common—drunkenness extremely prevalent.

*Venereal Affections* are very rare; nor can this arise, as at some stations, from any sanatory regulations to prevent the propagation of the disease, such precautions being unknown.

### 2. *Nova Scotia and New Brunswick.*

*The Peninsula of Nova Scotia,* as it is generally termed, extends in an oblong shape about 280 miles in length, and from 50 to 100 in breadth. Being bounded on the south-east and south by the Atlantic, on the west by the Bay of Fundy, and on the north by Cape Breton and a portion of the

Gulf of St. Lawrence, its position is perfectly insular, except where a strip of land, only eight miles in breadth, connects it on the north-west with the province of New Brunswick. The superficial area of this peninsula has been computed at 15,600 square miles, of which, however, not a twentieth part is cleared or under cultivation. Its physical aspect, like that of all the settlements in British America, is by no means mountainous; a chain of hills extends along the western side, from which a loftier range branches off to the north, but neither of them are of any great elevation, the highest point not exceeding 800 feet. In other parts the ground is diversified by numerous though slight undulations, and, except along the banks of the rivers and at the heads of some of the bays, level tracts of any extent are seldom to be met with.

The south-east coast, facing the Atlantic, is bold, rocky, and sterile, but at the mouths of rivers and creeks, occasionally intersected by small patches of alluvial soil.

The south and west coasts are deeply indented with numerous bays and lagoons by the action of the Gulf Stream, which, rushing upon this portion of the American continent in tides of from 60 to 70 feet, overflows the country to the distance of several miles, and converts the mouths of streams fordable at low water, into extensive arms of the sea where whole fleets might ride at anchor.

As a necessary consequence of this phenomenon, a very considerable portion of the banks of the rivers and the heads of the bays on that side of the peninsula are constantly in a marshy state, and as these tides are thickly impregnated with alluvial soil, owing to the force with which they are impelled against the adjacent continent, they leave on their reflux a considerable deposit of mud, which, by the aid of embankments, is converted into rich meadow land; and even where no such care has been taken, spontaneously supplies a coarse winter fodder for cattle.

The western coast and interior of the Peninsula appear to be rife in the causes of intermittents, yet the inhabitants enjoy an almost total exemption from them, and, indeed, a remarkable degree of health.

The climate of this province is distinguished by great and sudden alternations of temperature, such as, even in the changeable climate of Britain, appear almost incredible; though Nova Scotia is in this respect less remarkable than some of the North American stations, the thermometer has been known to exhibit a difference of 52° in 24 hours. The atmosphere is also exceedingly moist, the showers heavier and more frequent than in Britain, and fogs are common along the sea-coast throughout the year, but particularly in May and June, though they seldom extend any distance into the interior.

Though the winter is no doubt exceedingly severe, as compared with that of Great Britain, yet the cold is not by several degrees so intense as in that part of the American continent further to the west, neither is the heat of summer so great, probably attributable to the insular situation of the province having a tendency to modify both these extremes; the thermometer is seldom lower than 6° or 8° below Zero in winter, or above 88° in summer, but we possess no table sufficiently accurate to exhibit its precise range for a series of years.

The prevailing winds are from the east in spring, from the south or southwest in summer and autumn, and from the north or north-west in winter, at which period a change to any other quarter is generally followed by a rapid rise in the thermometer, accompanied by much rain or snow.

From December to the end of March the ground is generally covered with snow. Summer follows winter in such rapid succession, that there is scarcely any spring, but the autumn is pleasant and sometimes of long duration, as towards its termination, there is frequently a continuance of what is termed the Indian summer, till December again ushers in the winter with its usual severity.

Such is the description which the Reporters offer of Nova Scotia. The military stations are Halifax, on the south-east coast—Windsor, Annapolis, and the adjacent islands of Cape Breton and Prince Edward. We have not space for the medical topography of these localities, suffice it to say, that though in many there are marshy grounds yet there is no evidence of the influence of malaria.

*The Province of New Brunswick* forms a section of the North American continent, extending between 45° and 48° north latitude, and 63° 47′ and 67° 53′ west longitude, and comprising nearly 14 millions of acres. Towards the north-east it is united to Nova Scotia by the narrow strip of land before referred to, but differs from that peninsula in being composed of bolder undulations, which in some parts assume a mountainous character; cultivation has also made less progress, the whole province, with the exception of a few spots along the banks of the principal rivers, being still covered with dense forests.

The troops are principally quartered at St. John's and Fredericton, both situated on a broad and extensive river, which intersects the province in a north-westerly direction. St. John's is built on a rugged rocky peninsula, where this river falls into the Bay of Fundy: the soil in the neighbourhood as well as along the sea-coast, to the distance of several miles, is dry and stony, and in the vicinity of the town tolerably well cultivated; but dense forests still occupy the back-grounds at a short distance from the river.

Fredericton, the head-quarters of the regiment in this province, lies about 80 miles higher up the river, on an alluvial strip of land about two miles in length, and so low as to be much subject to inundations. The streets during rainy weather are almost impassable, and the banks of the river generally wet and muddy. Cultivation has made very little progress in the vicinity, which is principally covered with dense forests of hard wood. The soil is either alluvial, mixed with gravel, or of a tenacious clayey nature, and by no means ready to absorb moisture. Notwithstanding the apparently unfavourable nature of the locality, both troops and inhabitants enjoy excellent health and a marked exemption from all diseases of a febrile nature.

The climate of New Brunswick, particularly at Fredericton, is not liable, during winter, to such sudden vicissitudes as that of Nova Scotia. The frost is steadier, and the winter more severe, as well as of longer duration; the heat of summer is also more intense, and the thermometer, in the course of one year, has been known to range from 96° above to 42° below zero, though these extremes are considerably beyond the usual average.

As in Nova Scotia, fogs are common along the sea-coast of New Brunswick, particularly in the months of May and June, but seldom extend any distance into the interior, nor do they appear in the slightest degree prejudicial to health, the stations most exposed to them being quite as free from sickness as any other part of the Command.

It appears, from tables furnished by the Reporters, that the sickness and mortality of the troops in Nova Scotia and New Brunswick, correspond closely with what is found to be the case amongst the Dragoon Guards and Dragoons of the United Kingdom. The annual ratio of admissions per 1,000 of mean strength in the Province is 820,—and in the United Kingdom 929. The annual ratio of deaths in the former is 14·7—and in the latter 14. Yet, in addition of the seemingly insalubrious climate of these American stations, the price of spirits is so extremely low as to admit of the soldier indulging frequently in intemperance, even on the limited amount of his surplus pay.

*Fevers.*—Almost all the fevers are of the common continued type which is found to prevail in every climate. Intermittents are so rare that not two cases occur among the whole force annually, and these have in almost every instance been traced to individuals who had previously suffered from them in Upper Canada. Though such an exemption might be expected for the troops at Halifax, owing to the arid, rocky nature of the soil, and the absence of all causes likely to induce that form of fever, yet it is singular that the same exemption should extend to the troops and inhabitants at Windsor, Annapolis, Fort Cumberland, and Fredericton—situations abounding with those supposed sources of malaria, which, in the provinces of Upper Canada, under a similar temperature, are found, or at least are supposed, to produce fevers of the remittent and intermittent type. On that subject the medical officer at Fredericton particularly remarks that, though the station is on the alluvial bank of a large muddy river, surrounded by forests interspersed with swamps, with vegetation in abundance, and in every shape undergoing decomposition, yet the diseases attributed to malaria are scarcely known; and the same subject is frequently adverted to with equal surprise by other officers who have served at that station and on the western coast of Nova Scotia, where the agencies supposed to generate malaria exist in such abundance.

This fact must be received, startling as it is. The induction, however, which lays intermittent fever to the operation of marsh miasma, is too strong to be shaken by many such exceptions. Some disturbing circumstance exists in this province which ought to be carefully sought after. That some will be ultimately found we cannot doubt.

*Diseases of the Lungs.*—The Reporters observe that, notwithstanding the severe winter and sudden atmospherical vicissitudes peculiar to Nova Scotia and New Brunswick, diseases of the lungs are less prevalent in the proportion of 125 to 148, and less fatal in the proportion of $7\frac{1}{10}$ to $7\frac{7}{10}$. It is true the results for the United Kingdom embrace a period of seven years only, while those of Nova Scotia and New Brunswick extend over twenty years; but, even if the same period is taken for both, the admissions will be found lower in the proportion of 120 to 148, and the deaths in the proportion of $7\frac{5}{10}$ to $7\frac{7}{10}$. Were any other proof necessary to show how little even the most acute of these diseases seems to be aggravated by such atmospheric influence, it would be found in the relative proportion treated for inflammation of the lungs and pleurisy in this Command, compared with the Mediterranean and Bermuda—climates remarkable for their mild and equable temperature.

*Diseases of the Liver.*—The prevalence of this class of diseases is almost the same as among the Dragoon Guards and Dragoons serving in the United Kingdom, and the mortality is only half as high. This fact tends to throw very considerable doubt on the supposed influence of spirituous liquors in inducing affections of the liver, at least in a cold climate; for, owing to the low price of these in Nova Scotia and New Brunswick, there are few stations where intemperance is carried to a greater extent; yet not only do the troops suffer less from diseases of the liver than at home, but the proportion of deaths is only two-thirds as high as among persons insured in the Equitable Office, who, from their rank in life as well as the caution exercised in their selection, are by no means likely to be addicted to that vice.

There seems to us a fallacy in the latter observation. Soldiers are men in the prime of life, selected from the healthiest. Insurers are by no means in the prime of life, and whatever "caution" may be used, are *not* by any means of the healthiest. The soldier is invalided for chronic disease that *will* prove fatal bye and bye—the insurer sticks to the office. We must be excused for remarking that we fear this is not the only medical fallacy involved in these Reports. The civilian or the mere soldier who begins to *reason* on diseases, is not unlikely to make blunders. And if those blunders are founded on figures they will probably prove all the worse.

*Epidemic Cholera.*—The following statements will be found as usual to support both the contagious and anti-contagious hypothesis of the origin of this disease.

The troops in this Command escaped this disease in 1832, when it raged with great severity in Canada, but in July 1834, it broke out among those at Halifax under the following circumstances.

On the 20th of that month a vessel from Quebec, where the cholera was then prevalent, entered the harbour of Halifax. During the voyage the crew had suffered severely from bowel complaints, and one of them was admitted into the poor-house labouring under symptoms of cholera, of which he died. About a week afterwards another fatal case occurred in a person occupying the same ward, and by the 7th August the disease began to be very general among the inmates of the establishment. The first cases were observed in the town about the 10th of August, from which period till the 24th the epidemic made rapid progress, and continued with various degrees of intensity till the end of September. The extent of its ravages cannot be accurately ascertained, but it is supposed that throughout the town and suburbs about 600 died. The number admitted into the civil hospitals was 1,020, and the deaths 382. The infirm, the drunken, and the dissipated were its principal victims, though to this there were many exceptions.

Among the military, two cases of simple cholera had been noticed in the 96th Regiment, on the 24th and 31st July, but it was not till the 8th of

August that the first fatal case occurred. After that period it spread throughout the garrison; the Rifle Brigade suffered most, indeed, to such extent, that 18 deaths took place between the 21st and 25th of August. The corps was, in consequence, sent to Sackville, about 8 miles from Halifax, after which only four new cases occurred. The success of this experiment led to the same measure being adopted with the 96th and 83rd Regiments, who were removed to an encampment in the vicinity of the town, with the like good effect; the disease ceased both among the civilians and military about the end of September, though a few isolated cases continued to present themselves for some weeks after.

During the whole of this period bowel complaints of various kinds were exceedingly common, even among those who escaped the graver forms of the disease.

Though the circumstances under which the disease first appeared were such as to favour the idea of contagion, yet nothing occurred in the course of its progress to strengthen that supposition; and neither the medical officers, nor those in immediate attendance on the sick, suffered in a greater proportion than persons not so exposed.

Of 293 women attached to the different corps, 37 were attacked and 16 died, being almost exactly the same proportion as among the soldiers. Children were remarkably exempt, for of 560 in the garrison only 16 were attacked, and 6 died. The officers also suffered but little; out of a strength of 60 only 4 were attacked, all of whom recovered.

### 3. *Canada.*

The Provinces of Upper and Lower Canada are included in this Command, of vast extent, and embracing great varieties of surface and of climate. It is impossible for us to do more than just to hint at them.

*The Province of Lower Canada* lies between 45° and 52° North Lat. and 57½° and 80° West Long., and may be said to extend from the shores of the Atlantic to 55 miles beyond Montreal, comprising an area of nearly 260,000 square miles. Of these 3300 are covered by lakes and small rivers, and 52,000 by the extensive surface of the St. Lawrence; leaving upwards of 200,000 square miles of land territory, of which not more than a fortieth part is under cultivation, the rest being still in its primitive state, covered with forests and over-run with dense vegetation of every description.

The climate of the lower province is distinguished for the extreme severity of its winter, and the sudden alternations of temperature to which it is subject; so remarkable are these, that at Quebec the thermometer has on some occasions been known to fall 70° in the course of 12 hours; the cold weather sets in as early as November, and from the end of that month till May, the ground remains covered with snow to the depth of 3 or 4 feet. During this period there is generally a clear dry atmosphere, with light north-westerly winds or calm weather, and the sensation of cold is by no means so acute as might be anticipated from the low range of the thermometer; but when the wind blows from the N. E., especially with any degree of violence, the intensity of the frost becomes so excessive, that on several occasions the mercury, frozen in the thermometer, has no longer served to record the extreme reduction of the temperature. The general range, however, during winter, is from the freezing point to 30° below zero.

Though there is seldom a complete thaw in winter, yet occasionally, when the wind changes to the southward and eastward, the usually clear and serene sky becomes overcast, the atmosphere damp, and a considerable rise in the thermometer takes place, accompanied by thick fogs and heavy falls of snow. The seasons do not glide imperceptibly into each other as in more temperate regions; summer succeeds so rapidly to winter that the thermometer sometimes rises to 80° at mid-day before the ground is clear from its covering of snow.

Summer commences about the middle of May, and is usually ushered in by moderate rains and a rapid rise in the temperature, though the nights still continue cool; but during June, July, and part of August, the heat is great, indeed often as oppressive as in the West Indies, the thermometer frequently attaining to 95° in the shade; in these months the rain is never excessive nor of long duration; the earth, already saturated by the melting of the snow, requires no further supply of moisture to aid the rapid progress of vegetation.

*Upper Canada* may be said to extend from the Hudson's Bay territory, in latitude 46° on the north, to that of the United States, latitude 42° on the south; and from the river Ottawa and boundary of Lower Canada on the east, to the distant shores of the Pacific.

A province of such extent as Upper Canada necessarily presents considerable diversity of climate, the intensity of the cold and duration of the winter gradually diminishing from the lower extremity of the province along the borders of Lakes Ontario and Erie. About Amherstberg, at the head of the latter, the climate is said to be much the same as in Britain, except that the extremes of cold and heat are greater; while in proceeding northward from the shores of these lakes, even the distance of 60 or 70 miles is found to produce a marked increase, both in the severity of the winter and its duration. Compared, however, with Lower Canada, it is, in general, shorter by at least two months, while spring and autumn are lengthened in a corresponding proportion. The frost is, perhaps, for a few days as intense, but never of such duration; and thaws occur at short intervals. Snow seldom falls in any quantity till towards the end of December, and usually disappears in March; and the severity of the winter is said to have perceptibly diminished of late years, as the surface has been cleared and brought under cultivation.

The climate is generally more equable than that of the lower province; and though the thermometer ranges higher by a few degrees, the summer heat is not so oppressive, probably owing to the influence so vast a body of water is likely to exert in modifying the temperature, and to the cooling breeze which prevails along the lakes during the day.

*Condition of the Barracks and Hospitals.*—The Reporters observe that the condition of the barracks and hospitals has been a frequent subject of complaint by the medical officers, particularly as regards the state of repair, and the perishable nature of the materials of which these buildings have been constructed. We have adverted to this circumstance, because in such a climate it is obvious that the most complete accommodation is absolutely requisite to protect the soldier against the inclemency of the weather. The space allotted to him in barracks, too, is described as being in many instances inadequate for healthy respiration. By some recent measurements, supplied by the officers of the medical department, the maximum allotted to each soldier is calculated at 300 cubic feet, while the minimum does not exceed 216 cubic feet, being little more than half what is deemed essential to health in the prisons of this country.

In all other respects the soldier would appear to have no reasons for complaint.

*Sickness and Mortality.*—It appears that among every thousand soldiers serving in Canada 1097 cases of sickness have occurred in the course of the year, being 168 per thousand more than among the Dragoon Guards and Dragoons serving at home. The deaths, as stated in the Medical Returns, have averaged $16\frac{1}{10}$ per thousand of the force annually, which is a medium between the ratio in Infantry Depôts and Cavalry Corps serving in the United Kingdom.

This calculation, however, applies only to the mortality caused by disease, as reported by the medical officers. The deaths stated in the War Office Returns, which include those from all causes, furnish very different results. Those returns make the average ratio of mortality about 20 per thousand annually, which would lead to the conclusion, that the climate of Canada operated much more prejudicially to the health of the troops than that of their native country; but on investigating the causes of this mortality, the greater number of the deaths omitted in the Medical Returns are found to have arisen from the vices and acts of the unfortunate victims themselves, or from circumstances in no way connected with climate. It is remakable, for instance, that no less than 122 deaths are ascertained to have taken place by drowning, exclusive of those in the Artillery, which would probably swell the number to 150, or about an eighth of the total mortality. Most of these men lost their lives in attempts to cross rivers or creeks for the purpose of deserting, a crime of very frequent occurrence throughout this Command, particularly at stations in the vicinity of the United States.

The Upper Province would seem to be healthier than the lower. The principal diseases are fevers, diseases of the stomach and bowels, diseases of the lungs, wounds and injuries, abscesses and ulcers, and the venereal.

*Fevers.*—The annual ratio of admissions for fever per 1000 of mean strength have been 214—of deaths, 2·4. These results exhibit a remarkable prevalence of fevers in Canada compared with what has been observed in Nova Scotia or New Brunswick, the proportion of admissions being at least thrice as high; this feature may be said to extend over every form of the disease, but particularly the intermittent, of which we find several thousand admissions recorded in the former, while scarcely one indigenous case has ever been known in the latter Command, though the general features and climate are in many respects so similar.

The localities most subject to the disease are in Upper Canada. The prevalence of intermittent fevers in Upper compared with that in Lower

Canada, is as 178 to 26. It is necessary, however, to keep in view that all the admissions from intermittent fever in Lower Canada did not originate there, by far the greater proportion of them having occurred among soldiers who came from the upper provinces while labouring under that disease, or who had acquired a predisposition to it during a previous residence there. Indeed, except at Isle aux Noix and the other small stations along the banks of the Richelieu, fevers of the intermittent type are rarely indigenous in Lower Canada; at Quebec they are said to be unknown, and at Montreal nearly so.

In Upper Canada these diseases prevail most among the troops stationed along the course of the great lakes from Kingston to Amherstberg; they are almost unknown at Penetanguishene and By Town. The settlers who reside even at the distance of a few miles inland rarely suffer from them; yet the districts enjoying this exemption are in many parts covered with lakes, intersected by streams, and abound in marshy ground, decayed vegetation, and all the other agencies to which the origin of this type of fever is generally attributed.

These diseases, too, are said to be comparatively rare wherever the surface is covered with dense forests, even though the ground is wet and marshy. The vicinities of lands recently cleared are most subject to them, particularly meadows or open patches of the forest, which, though denuded of trees, have not been brought under cultivation; it would appear, too, that their prevalence is diminishing with the progress of agricultural improvement; for it will be observed on reference to the Abstract of Diseases, No. III. of Appendix, that since 1831, a period during which this province has been rapidly advancing in wealth and population, and many important changes have taken place in the vicinity of the stations occupied by the troops, intermittents have become comparatively rare, the proportion attacked having been scarcely one tenth part so high as the average previous to that period.

The febrile diseases of Upper Canada are by no means uniform in their prevalence; even in years when the degree of temperature, fall of rain, or extent of vegetation, have been much the same, the proportion of cases, particularly of intermittents, is very different. A general impression exists that their prevalence is in some measure dependent on the height of the waters in Lake Ontario, which attain their maximum in June or July. But the Reporters shew by statistical returns that such is not really the case.

Though there exists but little intermittent fever in the lower provinces, the common continued form is very prevalent, about 131 per thousand of the force being attacked by it annually; this is one-half more than the proportion in Upper Canada, and nearly twice as much as in the United Kingdom or the maritime provinces of Nova Scotia and New Brunswick. Owing to this circumstance, though fevers are on the whole more prevalent in the upper province, they cause exactly the same degree of mortality as in the lower, the greater number of cases in the former being counterbalanced by the more fatal character of those which appear in the latter.

*Diseases of the Lungs.*—Though the degree of mortality is much the same the proportion of admissions from this class of diseases is greater than in Nova Scotia and New Brunswick, principally owing to the more general prevalence of inflammation of the lungs and catarrhal affections. This peculiarity does not extend, however, over the whole of Canada, but is principally confined to the lower province.

"The following results afford another interesting proof how little the tendency to consumption is increased, either by intensity of cold, or sudden atmospherical vicissitudes:—

| | Aggregate Strength at each Station. | Total Attacked by Consumption. | Ratio per 1000 Attacked Annually by Consumption. |
|---|---|---|---|
| Bermuda for 20 years ... | 11,721 | 103 | 8.8 |
| Gibraltar for 19 years ... | 60,269 | 394 | 6.5 |
| Canada for 20 years .... | 61,066 | 402 | 6.5 |

In the two former climates, the thermometer rarely falls to the freezing point, and its fluctuations are comparatively trifling; in the latter, the cold is often so intense as to freeze mercury, the variations sometimes exceed 50° in the course of a few hours, and the soldier in passing from his heated guard-room to his night-duties in the open air is not unfrequently exposed to an immediate change of temperature exceeding 100°."

*Epidemic Cholera.*—A very circumstantial and interesting account is given of the progress of this epidemic. The conclusion, perhaps, contains the pith of the matter.

"These circumstances, combined with the fact of several persons having died from the disease on their passage from Ireland, in each of the years when it appeared, led to the belief of its having been imported and subsequently communicated by contagion; various precautionary measures were in consequence adopted to prevent its propagation, and strict quarantine regulations were enforced, both as regarded the troops and inhabitants; but though in some instances these were apparently effectual, in others they proved of little avail, and the contagious nature of the disease was subsequently rendered extremely questionable from the circumstance, that neither the physicians nor those in constant attendance on the sick, exhibited any peculiar liability to it.

Of course it is impossible, in a limited report of this nature, to enter fully on all the facts and arguments bearing on the important and much-disputed topic of contagion; we can only say that all that has been adduced on either side seems to fall far short of absolute proof, and even those who have had the best opportunities of forming accurate opinions, by watching the progress of this disease, are forced to admit that its origin is still involved in mystery, or at least, that the contrariety of results can only be reconciled by supposing that under some circumstances it may be contagious, while in others it may be the reverse."

The following statement is not flattering to physic, yet, we think, not far from the truth.

"One of the most extraordinary features of this epidemic is, that the proportion of deaths to recoveries has been very nearly alike, in all the military Commands of which the medical records have been investigated, for instance:—

| | Attacks. | Deaths. | Proportion of Deaths to Attacks. |
|---|---|---|---|
| Among Cavalry in the United Kingdom 1832, 1833, and 1834.............. | 171 | 54 | 10 in 32 |
| „ Troops in Gibraltar, 1834 ...... | 459 | 131 | 10 „ 35 |
| „ „ Nova Scotia, &c. 1834 | 210 | 59 | 10 „ 35 |
| „ „ Canada, 1832 ........ | 259 | 94 | 10 „ 28 |
| „ „ Canada, 1834........ | 97 | 33 | 10 „ 29 |
| „ Black Troops at Honduras, 1836 | 62 | 20 | 10 „ 31 |

Thus, under all the modes of treatment which may have been adopted on these different occasions, the proportion of deaths to recoveries has not varied above one-fourth, showing that the remedial measures hitherto employed can have had little if any effect in counteracting the fatal character of the disease.

In both these years, when this epidemic prevailed, the native Indians suffered from it to the same extent as the white population. At three settlements from which returns were received, about a twelfth part of the population died in 1832, and about half that proportion when it again prevailed in 1834. Although their principal remedy consisted in swallowing large quantities of charcoal mixed with lard, almost exactly the same proportion recovered as among the white inhabitants of the towns, who possessed every advantage which the aid of medical science could suggest."

It would appear, from statements of this description, that physic had little influence on the disease. We believe this to be far from the fact, and the next paragraph will explain our meaning.

"Not a single officer died, and only four were attacked during the first, and three during the second epidemic. The same peculiarity was observed during the prevalence of this disease in Nova Scotia, in 1834; and in Gibraltar there were but two admissions and one death among the officers, though there were 459 admissions and 131 deaths among the troops. This leads to the inference that though little can be done to ameliorate the character of the disease when allowed to arrive at an advanced stage, yet that a generous diet, regular habits, and the degree of attention which persons in the higher ranks of life are likely to pay to its premonitory stages, have a powerful effect in diminishing their liability to its influence."

In fact we are of opinion that physic can save many lives in an outbreak of epidemic cholera. The bad cases are not cured but may be prevented by it, and more especially by the treatment of the premonitory diarrhœa. Of this we have been satisfied by personal experience, as well as by the experience of others.

*Delirium Tremens.*—In Nova Scotia and in Canada, there is a similar prevalence of *delirium tremens* (resulting from intoxication), with the exception that in Canada the cases have become less numerous and less fatal since 1832, indicating, it is to be hoped, a diminution in the vice of intemperance, whereas in Nova Scotia they appear to be on the increase.

*Rheumatic Affections.*—It is a striking circumstance, that in a climate of which the temperature in winter is so low, and liable to such sudden alternations as that of North America, the proportion of rheumatic affections should be less than in the United Kingdom. It is even lower than in the Mediterranean or Bermuda, though there the thermometer rarely falls to the freezing point, and the atmospherical vicissitudes are comparatively trifling. Observations recently made by the Medical Association of Great Britain on the prevalence of rheumatic affections in different countries of this kingdom have given similar results, and established that these diseases are less under the influence of atmospheric agency than has generally been supposed.

*Venereal Affections* are only about half as prevalent as in the United Kingdom; and a reference to the Abstract of Diseases, No. III. of Appendix shows, that in Canada syphilis has of late years become almost extinct, not more than three in every thousand soldiers having been treated for it annually, and of these the greater proportion were recruits who had contracted the disease in this country. So far as we can learn, there are no sanatory regulations to prevent its propagation, but probably at the remote stations opportunities of contracting it are comparatively rare, to which circumstance also may be attributed the marked exemption of the detachments in the upper provinces, among whom cases are seldom or never observed.

### 4. *Newfoundland.*

This island lies on the north-east side of the Gulf of St. Lawrence, and adjacent to that part of the American continent termed the coast of Labrador. Its extreme length is 420 miles, and extreme breadth 300; but of this extensive territory the greater part is as yet unexplored. The few tracts visited by Europeans have been found hilly, and sometimes even mountainous, varied by extensive plains covered with grass or low stunted bushes, and intersected by numerous rivers and lakes. The forests are not so dense, nor the timber of such magnitude as in the Canadas, both soil and climate being less favourable to vegetation.

Towards the south-east extremity, the deep bays of Trinity and Placentia intersecting the island in opposite directions cut off a peninsular section about 78 miles in length, and 75 in breadth, where the British population principally reside in several small towns and villages along the shores of the numerous bays by which that portion of the island is indented.

One of these bays between two high mountains on the east coast, and having a very narrow entrance protected by extensive fortifications, forms the harbour of St. John's, on the shores of which stands a town of that name, the capital of the island. In its vicinity are three forts where the troops are quartered.

The climate of the southern portion of Newfoundland is similar to that of Nova Scotia, except that the summers are colder, of shorter duration, and liable to more sudden vicissitudes, owing to the melting of the icebergs on the coast, which exerts considerable influence on the temperature; the island has also been long noted for the frequent and dense fogs which prevail along its banks, and often continue during a great part of the summer. None of these agencies, however, seem to operate prejudicially on the health of the inhabitants, among whom the mortality is on a lower scale than in any portion of the American continent.

From a return of the population, births, and deaths for the years 1822, 1825, and 1827, it appears that the population was 55,001—the average of births 1,785,—and the average of deaths 724.

"According to this table the deaths are only 1 in 76 of the population—an exceedingly low ratio indeed, especially when it is considered that upwards of 20,000 are children under 15 years of age. As the inhabitants are scattered over a great extent of coast, several of the deaths may possibly have been omitted; but, even making all due allowance for that source of error, their rapid increase, without any material aid from immigration, furnishes sufficient proof that the climate, however unpleasant to the feelings, is highly favourable to the constitution.

Had we drawn our conclusions in regard to the climate, however, from the mortality among the troops at this station, we should have been led to very different conclusions."

It is only since 1825 that available returns have been obtained from the troops. Since that year a corps has been formed for service in this colony, consisting of three companies of Veterans, who, although for the most part aged or disabled, have been reported as fit for garrison duty. These, with a company of Artillery, have generally constituted the whole force.

From a table it appears that the mortality among the Veterans has been upwards of 41 per thousand annually on the average of the last 12 years, while that of the Artillery has been only 22 per thousand during the same period. The high ratio among the former may in part be accounted for by their advanced age, nearly one half being between 33 and 40, and the other half above that period of life; but it appears still more attributable to the immediate effects of intemperance, as the records of that corps furnish most startling evidence of the general prevalence and destructive consequence of this vice.

Of 100, the total, deaths in the Veteran Companies, from 1825 to 1832 inclusive, 42 were the *direct* effects of intoxication! When to this we add indirect destruction produced by other diseases, to which such drinking must have lent a helping hand, we have a very pretty commentary on the system of strong potations.

The Reporters add :—" The fate of so large a proportion of this garrison, by their own imprudence in the use of spirituous liquors, affords a striking illustration of the progressive effect and ultimate consequence of long-continued habits of intemperance. In Nova Scotia, for instance, we find that, though this vice prevails to a great extent among the troops, the mortality is as low as can be expected in any climate, even among persons of abstemious habits. But there the troops are, for the most part, men in the prime of life whose excesses produce little sickness or mortality, while they have the advantage of youth on their side; but they are silently laying the seeds of disease in their constitution, and inducing premature old age and disability, so that by the time they attain the same advanced period of life as the Veterans, a repetition of excesses, which might formerly have been indulged in with comparative impunity, hurries them to an untimely grave.

In regiments of the line the number of men at an advanced period of life being but small, the premature deaths caused by drunkenness are lost in the mass, and, as we have frequently stated, add little to the general mortality. It is only when a corps is composed of men advanced in years that the ultimate consequences of this vice can be traced to their full extent, or so strikingly manifested as in the present instance."

It is obvious that, in order to form an opinion on the salubrity or otherwise of foreign stations, the amount of *invaliding* must be taken into the account.

Subsequent to 1824, and exclusive of 313, who, after being sent home invalided, returned fit for further service to their depôts or corps, the proportion discharged has amounted to 19 per thousand annually, being the average of the Mediterranean stations, and nearly the same as among the Dragoon Guards and Dragoons serving in the United Kingdom. These returns apply to the whole of British America, including Bermuda, Nova Scotia, the Canadas, and Newfoundland.

There is one important statement in reference to pulmonic affections. In British America, the ratio per thousand invalided annually for pulmonary affections has been $3\frac{9}{10}$—in Gibraltar $3\frac{9}{10}$—in Malta 5—in the Ionian Islands $2\frac{8}{10}$.

"It is thus manifest that in the mild climate of Malta one-fifth more are invalided for pulmonic affections than in British America—a sufficient reason, combined with what has previously been stated on the subject, to render extremely questionable the generally received opinions in regard to the influence of temperature in inducing or aggravating these diseases."

### *Greater Mortality in advanced Life in North America.*

In all the North American Commands mortality increases with the advance of age much more rapidly than in the United Kingdom or Mediterranean. This is particularly observable in the Bermudas, where, though the ratio between the ages of 18 and 25 is much the same as among the Dragoon Guards and Dragoons in this country, it is, at the subsequent periods of life, more than twice as high.

So far as regards the Bermudas this may partly be accounted for by the circumstance, that in all warm climates the constitution appears to deteriorate rapidly with the advance of age. This will not, however, account for the same feature being manifested, though in a less striking degree, on the continent of North America; perhaps the repeated attacks of febrile diseases to which the troops in Upper Canada are subject as well as the general prevalence of intemperance, though not in themselves an immediate source of mortality, may, by tending to sap the vital energies, and inducing premature old age, ultimately lead to this peculiarity.

This corroborates the remark we made a little way back, that the seeming impunity with which ordinary causes of disease have been applied to troops, must be taken with reservation. They are resisted in the hey-day of life, but they materially curtail its duration. It is obvious that two men may pass through the period of service, and each leave the army at the same time—the one with a constitution shattered and a few years only of life before him—the other hale and enjoying the ordinary probabilities of longevity. Such Statistical Returns as those before us take small account of this, a most important element in calculating the influences of climate.

*Deductions from the Preceding Report.*

*a. Pulmonary Affections.*—" Having, in the course of this Report, frequently adverted to the uniform degree of prevalence which, notwithstanding the dissimilarity of climate, has been found to exist in the proportion of pulmonary affections in Nova Scotia and Canada, compared with Malta and Gibraltar, it seems unnecessary here to recapitulate the evidence on that subject; but it may be proper to inquire whether there exists, in the moral or physical condition of the troops in the Mediterranean and American stations, any difference likely to have influenced the results on which that comparison has been founded.

In the last section of the West India Report we showed that these diseases, even under the high temperature of the tropics, prevailed to a greater extent than in the United Kingdom. But it may be argued that several circumstances, independent of climate, were there in operation to induce that peculiarity; for instance, the innutritive qualities of the diet, the limited and defective state of the barrack accommodation, and the general prevalence of intemperance, were all causes tending to affect the health of the troops in no inconsiderable degree. We are, therefore, led to inquire whether any deteriorating circumstances of a similar nature exist in the Mediterranean from which the troops in North America are exempt, and by which the tendency to these diseases may have been so far aggravated as to counterbalance the advantages otherwise resulting from its mild and equable climate.

So far, however, from this being the case, every circumstance has been more favourable to the troops in the Mediterranean. The barrack and hospital accommodation, in Malta and Gibraltar at least, is not only of a more substantial nature than in Canada and Nova Scotia, but nearly double the space is allotted to each soldier; the diet, with the exception of a greater issue of salt meat in Gibraltar during the winter months, is nearly the same, and the meals are regulated on similar principles. Intemperance, to which so much has been attributed as an exciting cause of these diseases, cannot be said to prevail to a greater extent in the Mediterranean than America, where the constant use of ardent spirits is likely to prove still more prejudicial than the low wines which form the principal medium of intoxication in the Mediterranean.

In all these respects, then, the troops in the Mediterranean have decidedly the advantage. We have yet to advert to another circumstance no less favourable to them: consumption, the most fatal of this class of diseases, is supposed to affect persons at an early period of life more than those of mature age. Now, owing to the frequency of desertion in North America, so many recruits have to be sent out from this country that nearly one-half of the force there is under 25 years of age, while in the Mediterranean, where no such necessity exists for large drafts from the depôts, the proportion under that period of life is only from a third to a fourth of the whole; consequently the composition of the force in the Mediterranean renders it much less subject to the influence of consumption, if not also of the other pulmonary diseases which frequently precede it.

When we find, notwithstanding all these circumstances apparently so favourable to the greater development of these diseases in Canada and Nova Scotia, that the troops there do not suffer from them to a greater extent than in the Mediterranean, it would manifestly be incorrect to attribute their prevalence in North America to the reduced temperature and sudden atmospherical vicissitudes incident to that quarter of the globe, seeing that the sufferings of the troops from these diseases are equally great in other climates where no such causes are in operation to induce them."

We would hint that the circumstance of so many young recruits being sent to North America, is by no means adverse to immunity from consumption. That disease is more rife at an early age it is true, and were persons at that age taken *indiscriminately*, there would be some force in the reporter's argument. But the recruits are *picked* young men, especially selected for that physical constitution which is indisposed to phthisis. North America then has numerous fresh draughts of the non-consumptive. Climate does not shew the whole evidence of its work, because the old hands *bolt*—and it cannot knock down, at all events at once, the hardy novi homines. We repeat that these Returns and Captain Tulloch's medical observations must be taken with reservation.

*b. Rheumatism.*—" The caution necessary to be exercised in attributing to certain peculiarities of climate the prevalence of any class of diseases, is so strikingly exhibited by the proportion of Rheumatic affections ascertained to have occurred among the troops in different colonies, that the following abstract will best serve to illustrate our observations on this head :—

| | Jamaica. | Nova Scotia, and New Brunswick. | Bermudas. | Malta. | Ionian Islands. | Gibraltar. | Canada. | Mauritius. | Windward and Leeward Command. | United Kingdom. | Cape of Good Hope. |
|---|---|---|---|---|---|---|---|---|---|---|---|
| Admissions from Rheumatic Affections annually per thousand of mean strength ........ | 29 | 30 | 33 | 34 | 34½ | 38 | 40 | 46 | 49 | 50 | 57 |

Thus we find that in the mild and equable climate of the Mediterranean or the Mauritius, the proportion of rheumatic affections is even greater than in the inclement regions of Nova Scotia and Canada, and that, though some of the provinces of the Cape of Good Hope have occasionally been without rain for several years, these diseases are more frequent in the dry climate of that Command, than in the West Indies where the condition of the atmosphere is as remarkably the reverse; yet have extreme cold and atmospheric vicissitudes, coupled with excess of moisture, been assigned as satisfactory causes for their prevalence." 45.

*c. Marsh Miasma and Fever.*—The results of this Report, in regard to the relative prevalence at different stations in British America of remittent and intermittent fevers, add still further to the difficulty of establishing any uniform connexion between the presence of marshy ground, and the existence of those febrile diseases to which the exhalations from it are supposed to give rise. As it was formerly shown that, in some of the Ionian Islands, totally destitute of marsh and comparatively barren of vegetation, more remittent and intermittent fevers have been under treatment among the troops, than in others where these alleged sources of disease existed in the greatest abundance; so in the present Report we find it established, that yellow fever of the most aggravated form has repeatedly made its appearance at Ireland Island in the Bermudas, a rocky barren spot only a few hundred yards in breadth, containing no marsh, and with little or no vegetation except a few cedar-trees.

Conversely, again, we find that these diseases prevail to a remarkable extent along the banks of the lakes, and the margin of the streams in Upper Canada, while they are comparatively rare in similar situations in the Lower Province; that among the troops at Fredericton, living on the marshy banks of a river, surrounded by dense vegetation, scarcely a case of them is ever known, and that a similar exemption is enjoyed even by those at Annapolis and Windsor in Nova Scotia, though quartered at the embouchure of rivers daily subject to extensive inundations, and of which the banks, for the distance of several miles, exhibit that combination of mud, marsh, and decayed vegetation, which is generally supposed a most prolific source of febrile diseases.

When, in subsequent Reports, we come to investigate the operation of these diseases, on the West coast of Africa and other colonies, we shall be able to adduce still more satisfactory evidence on this subject; in the mean time, we have felt it our duty to place the preceding facts in a prominent point of view—not for the purpose of establishing any particular theory, but to show how inadequate, in many instances, is the supposed influence of emanations from a marshy soil to account for the origin of these diseases. All the evidence obtained seems only to warrant the inference that a morbific agency of some kind is occasionally present in the atmosphere, which, under certain circumstances, gives rise to fevers of the remittent and intermittent type; and that though the vicinity of marshy and swampy ground appears to favour the development of that agency, it does not necessarily prevail in such localities, nor are they by any means essential either to its existence or operation.

*d. Removal of Stations for Escape.*—" Notwithstanding the doubt in which this branch of the investigation is still involved, we may venture, from the facts adduced in all the Reports hitherto submitted, also to draw the conclusion, that when this morbific agency manifests itself in the epidemic form, its influence is frequently confined to so limited a space as to afford a fair prospect of securing the troops from its ravages, by removal to a short distance from the locality where it originated. The history of the epidemic fevers at Gibraltar furnishes several remarkable instances of this kind, and we have also shown that, both in the West Indies and Ionian Islands, one station has frequently suffered to a great extent from yellow fever, while others within the distance of a few miles have been entirely exempt. In the epidemic cholera at Montreal and Halifax, which seems to have been in this respect somewhat analogous in its operation, we have also had occasion to remark the sudden cessation of the disease immediately on the removal of the troops even to a short distance.

